# Adding Diverse Noncanonical Backbones to Rosetta: Enabling Peptidomimetic Design

**DOI:** 10.1371/journal.pone.0067051

**Published:** 2013-07-15

**Authors:** Kevin Drew, P. Douglas Renfrew, Timothy W. Craven, Glenn L. Butterfoss, Fang-Chieh Chou, Sergey Lyskov, Brooke N. Bullock, Andrew Watkins, Jason W. Labonte, Michael Pacella, Krishna Praneeth Kilambi, Andrew Leaver-Fay, Brian Kuhlman, Jeffrey J. Gray, Philip Bradley, Kent Kirshenbaum, Paramjit S. Arora, Rhiju Das, Richard Bonneau

**Affiliations:** 1 Department of Biology, Center for Genomics and Systems Biology, New York University, New York, New York, United States of America; 2 Department of Biochemistry, Stanford University, Stanford, California, United States of America; 3 Department of Chemical and Biomolecular Engineering, The Johns Hopkins University, Baltimore, Maryland, United States of America; 4 Department of Chemistry, New York University, New York, New York, United States of America; 5 Department of Biomedical Engineering, The Johns Hopkins University, Baltimore, Maryland, United States of America; 6 Department of Biochemistry, University of North Carolina, Chapel Hill, North Carolina, United States of America; 7 Program in Molecular Biophysics, The Johns Hopkins University, Baltimore, Maryland, United States of America; 8 Fred Hutchinson Cancer Research Center, Seattle, Washington, United States of America; 9 Computer Science Department, Courant Institute of Mathematical Sciences, New York University, New York, United States of America; University of South Florida College of Medicine, United States of America

## Abstract

Peptidomimetics are classes of molecules that mimic structural and functional attributes of polypeptides. Peptidomimetic oligomers can frequently be synthesized using efficient solid phase synthesis procedures similar to peptide synthesis. Conformationally ordered peptidomimetic oligomers are finding broad applications for molecular recognition and for inhibiting protein-protein interactions. One critical limitation is the limited set of design tools for identifying oligomer sequences that can adopt desired conformations. Here, we present expansions to the ROSETTA platform that enable structure prediction and design of five non-peptidic oligomer scaffolds (noncanonical backbones), oligooxopiperazines, oligo-peptoids, 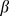-peptides, hydrogen bond surrogate helices and oligosaccharides. This work is complementary to prior additions to model noncanonical protein side chains in ROSETTA. The main purpose of our manuscript is to give a detailed description to current and future developers of how each of these noncanonical backbones was implemented. Furthermore, we provide a general outline for implementation of new backbone types not discussed here. To illustrate the utility of this approach, we describe the first tests of the ROSETTA molecular mechanics energy function in the context of oligooxopiperazines, using quantum mechanical calculations as comparison points, scanning through backbone and side chain torsion angles for a model peptidomimetic. Finally, as an example of a novel design application, we describe the automated design of an oligooxopiperazine that inhibits the p53-MDM2 protein-protein interaction. For the general biological and bioengineering community, several noncanonical backbones have been incorporated into web applications that allow users to freely and rapidly test the presented protocols (http://rosie.rosettacommons.org). This work helps address the peptidomimetic community's need for an automated and expandable modeling tool for noncanonical backbones.

## Introduction

A variety of peptidomimetic oligomers have been identified that can mimic protein secondary structure features and can exhibit many of the physiochemical properties of polypeptides, including the spacing and geometry of side chains [1]–[5]. Here we focus on several peptidomimetic scaffolds that enable the use of large libraries of potential side chains and are compatible with facile monomer or sub-monomer synthesis. Of particular importance is the fact that functional groups on peptidomimetics often have substantial spatial and geometrical congruence with side chains presented on protein secondary structure [6]. For example, peptidomimetics can be used to mimic large binding interfaces mediated by protein helices or strands [7]. The chemical identity of peptidomimetic side chains and termini can be tailored to establish proteolytic stability, membrane permeability, and additional desirable pharmacological properties. These characteristics may endow peptidomimetics with improved therapeutic potential relative to canonical peptide analogues. Specific examples relevant to this work include the development of protein interaction inhibitors and the antagonism of interfaces larger than those targeted by small molecules [8], [9]. Additional conformational diversity provided by some classes of peptidomimetics make them an attractive system for “foldamer” research with the goal of developing new secondary or tertiary structural motifs. Here we discuss five non-peptidic oligomer systems capable of addressing these goals: oligooxopiperazines, oligo-peptoids, 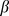-peptides, peptide hydrogen bond surrogate helices and oligosaccharides.

**Figure 1:**
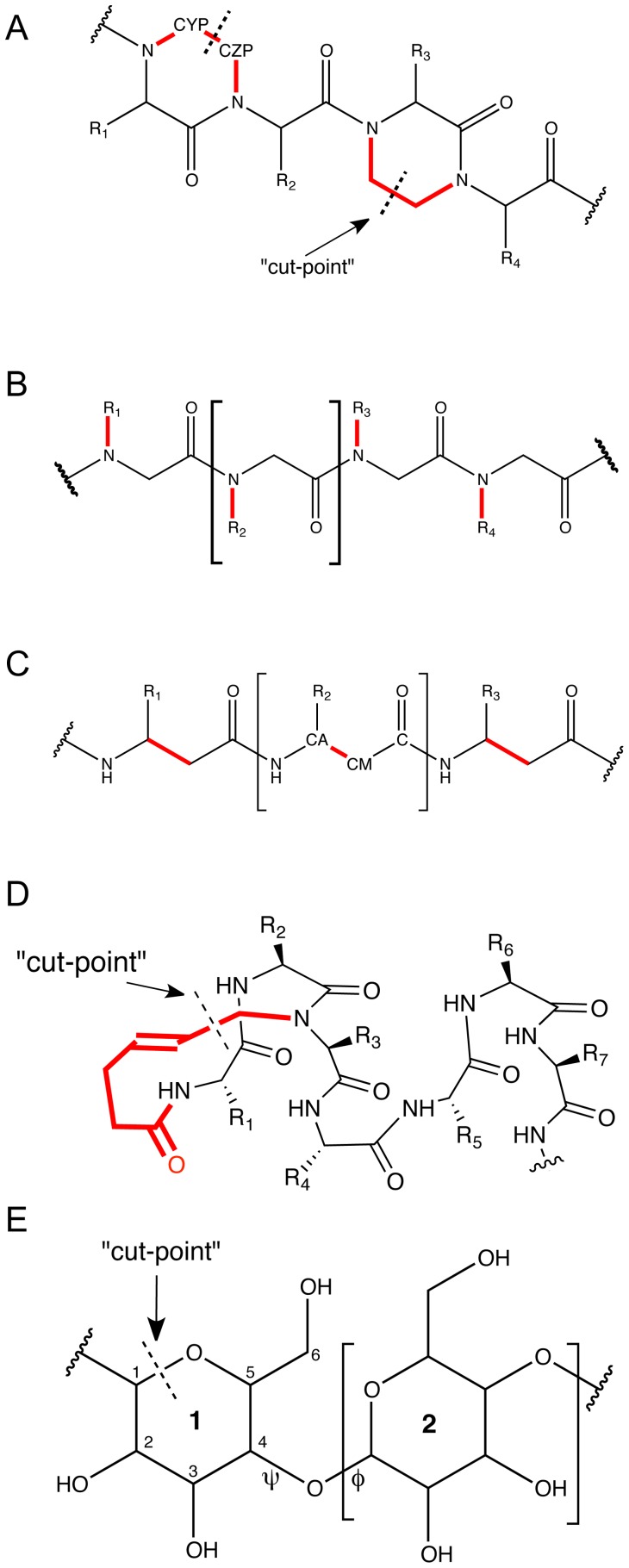
Chemical structures of noncanonical backbones in Rosetta. A) Oligooxopiperazines (OOP), B) Peptoids, C) 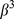-peptides, D) Hydrogen Bond Surrogate (HBS) helices, E) Oligosaccharides. In A–D, bonds highlighted in red deviate from the traditional peptide backbone. Arrows point to “cut-points” described in text. Brackets delineate a single residue subunit.

The oligooxopiperazine scaffold (OOP) (figure 1A) is a peptidomimetic with side chains that can mimic the *i*, *i*+4 and *i*+7 positions of an 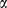-helix [10]. An OOP monomer is synthesized from amino acids with ethylene bridges linking neighboring pairs of backbone amides, resulting in a backbone composed of linked six-membered rings. Because 62% of protein complexes in the PDB include an 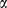-helix at an interface [7], helical mimetics such as OOPs serve as useful inhibitor scaffolds for many large protein interfaces [4], [11].

Peptoids (figure 1B) are oligomers of *N*-substituted glycine units in which the side chains are positioned on the backbone nitrogen (as distinct from the 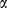-carbon) [12]–[14]. This results in an oligomer backbone that is achiral, proteolytically stable, and displays increased flexibility at its amide (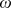) dihedral angle, exhibiting both *cis* and *trans* conformations [15]. The ability of the backbone amide to populate both *cis* and *trans* conformations allows peptoids to mimic diverse protein secondary structure features including both poly-proline type I and type II helices [16], [17]. Peptoid structures are being explored in the context of material science and biomedical applications [18]–[22].


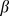
-peptides (figure 1C) are peptides with an additional backbone carbon resulting in an extra dihedral angle along the backbone and extended length between adjacent side chains. Several groups have used 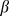-peptides as a system for foldamer research where the goal is to create protein-like or DNA-like structure and function with oligomers other than 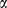-peptides and nucleic acids [23]–[25]. The Gellman lab, for example, has pioneered the creation of heterogeneous backbones, combining both 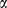 and 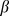 peptides in an alternating fashion to form helices. These molecules have been used as high affinity binders to the Bcl-xL family of prosurvival proteins [26] as well as gp41 inhibitors [27].

Hydrogen bond surrogate (HBS) helices (figure 1D) contain a carbon-carbon bond in place of the canonical *i* and *i*+4 backbone hydrogen bond in alpha-helices [28]. This covalent bond allows much shorter peptides to stably form an 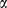-helix conformation. HBS helices have been shown to mimic protein interaction interfaces including those of p53 [29], HIF-1alpha [9], SOS [30] and MCL-1 [31].

Oligosaccharides (figure 1E) are chains of glycosidically linked monosaccharides, which preform diverse functions in the cell including cell-cell recognition [32]. Additionally, glyco-proteins (i.e. proteins that are covalently linked to saccharides) are involved in protein folding [33], cell signaling [34], [35] and when misregulated, prion diseases [36], [37]. Although oligosaccharides are not traditional peptidomimetics, their implementation into the ROSETTA framework provides an excellent example of the type of diverse backbones that can be implemented and modeled using this approach.

Currently many peptidomimetics are designed manually with no computational framework to efficiently search their available conformational and design spaces. Database mining tools are available to match potential inhibitor scaffolds to specific protein interaction interfaces but these tools lack the ability to explicitly model and redesign the scaffold [6]. This scarcity of modeling tools limits the progress of applying peptidomimetics to many applications for which they are attractive. Recent advances to the molecular modeling suite ROSETTA create a framework that allows the modeling and design of noncanonical backbones including the ones just described. The framework allows access to a variety of energy functions and algorithms provided by ROSETTA such as minimization, side chain packing/design and Monte Carlo conformational search. Important precedents for this work include prior efforts of ligand docking in ROSETTA [38], the incorporation of noncanonical side chains into ROSETTA [39] and work to derive methods for creating rotamer libraries for noncanonical amino acids (NCAAs) [39], [40].

**Figure 2:**
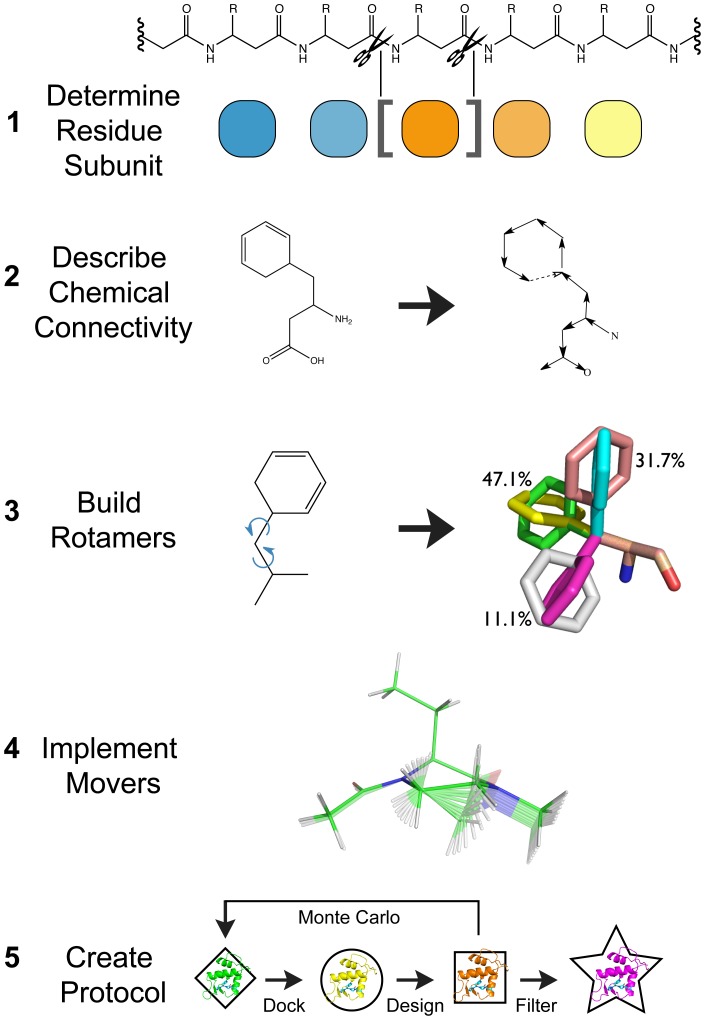
Noncanonical backbone implementation flow chart. Outline of the five steps needed for implementation of a noncanonical backbone into ROSETTA.

To take advantage of the algorithms and scoring functions available in ROSETTA, noncanonical backbones must have their chemical descriptions (the start and stop of each monomer) and kinematics (methods for altering conformations) instantiated properly within the framework. Here we describe the implementation of five noncanonical backbones (OOP, oligo-peptoids, 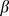-peptides, HBS helices and oligosaccharides) as working illustrations of a general approach for implementing noncanonical backbones in ROSETTA. We outline five steps (figure 2) to achieve this goal: 1) determination of the boundaries of each residue subunit from its backbone chemical structure, 2) description of the chemical connectivity within the ROSETTA framework, 3) building and parameterizing rotamer libraries, 4) implementing movers (kinematics) to sample conformations and 5) creation of overall modeling/design protocols (this last step will be variable depending on project goals; in this work we focus on design protocols for antagonists of large protein-protein interfaces as well as the redesign of 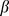-peptide helix bundles).

We also present new computational results, including comparisons of a ROSETTA score function to quantum mechanical calculations on the OOP backbone. Our target audience is primarily current and future developers interested in expanding ROSETTA to additional classes of polymers; casual developers interested in creating new ROSETTA protocols incorporating one or more noncanonical backbone positions or side chains; and non-experts interested in using these tools through the ROSIE (Rosetta Online Server that Includes Everyone) server (http://rosie.rosettacommons.org). All associated code is freely accessible to academic users via the ROSETTACOMMONS website (http://www.rosettacommons.org).

## Methods

The recent reorganization of the ROSETTA code (i.e. ROSETTA3) to comply with standard object oriented software practices has provided a flexible framework to model oligomers other than the traditional peptide and nucleic acid backbones [41] and also to model large heteromeric complexes involving mixed types of polymers (like protein/peptidomimetic complexes, see below). In this framework the residue object is the central object; all algorithms and scoring functions within ROSETTA act upon residues (whether amino acids, bases, peptoid monomers, etc.). A first step is to define the chemical structure of the repeating subunit in the noncanonical backbone, as a residue (described as a ResidueType object within ROSETTA). Generally, residues in ROSETTA are defined such that a subunit contains a single side chain with a corresponding rotamer set.

The next step after determining a residue subunit is to describe the subunit in terms of its chemical configuration in a format readable by ROSETTA (ROSETTA ResidueType). ROSETTA provides two ways to describe new ResidueTypes, the residue params system and the residue patch system, both based on easily edited text files placed in the ROSETTA database directory. The residue params system describes a ResidueType completely with all atoms and bonds as if it were fully connected to its neighboring residues. The fully connected ResidueType is called a mid or base variant and includes a unique residue name, atom descriptions (i.e. name, type, charge), bond connectivity (including to neighboring residues), and idealized internal coordinates. The residue params system is useful when defining polymer subunits that have limited similarity to existing ResidueTypes (see peptoid section).

Another way ROSETTA allows ResidueType declarations is using the patch system. This system defines new ResidueTypes based on previously declared ResidueTypes. For example, the CtermProteinFull patch is applied to all existing ResidueTypes (e.g. alanine) to define the C-terminal variants of standard residues (e.g. C-terminal alanine). Additionally, ResidueTypes may have multiple patches applied. For instance a C-terminal phospho-tyrosine is created by applying both a C-terminal patch and a phosphorylation patch. The patch description includes a unique patch name, a section defining which ResidueTypes to apply the patch to, and atoms to be added (or deleted) to applicable ResidueTypes. The residue patch system is useful when defining polymer subunits that are modifications of existing ResidueTypes and can be implemented with a single patch declaration (see OOP section).

After the new ResidueTypes are defined and readable by ROSETTA, it is necessary to create rotamer libraries. Backbone dependent rotamer libraries can be generated using the MakeRotLib protocol that samples side chain 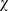 angles of an amino acid for 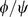 combinations of backbone angles. Side chain conformation samples are clustered and an energy score is calculated for each cluster. The energy is then converted into a probability that serves as an entry in the ResidueType's rotamer library. The full protocol is discussed by Renfrew et al. 2012, along with a protocol capture describing how to run the code [39]. It should be noted that it is unnecessary to create new rotamer libraries if it is believed there is a suitable available rotamer library. For example in the case of HBS helices the backbone is similar to the peptide backbone (identical at most positions) and thus protein/peptide rotamer libraries are used. This is often the case when making new ResidueTypes using the patch system. If the modifications made to the base ResidueType do not affect side chain degrees of freedom (or do not make other significant changes to the chemistry), the base rotamer libraries may be used for patched variants.

Once the noncanonical backbone chemical structure is fully described, kinematic functionality (specialized movers) can be developed to properly sample the conformational space specific to the backbone. Backbones with additional degrees of freedom, such as a flexible 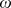 angle (e.g. oligo-peptoids) or an added backbone dihedral (e.g. 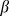-peptides), require the implementation of one or more new movers. It should be noted that the degrees of freedom sampled are dependent on the modeling goal. For instance, if appropriate modeling can be achieved using fixed backbone design, specialized backbone moves may not be necessary.

Finally, with both the chemical structure and kinematics defined, a full protocol can be developed that focuses on the specific modeling goals of the application. A full protocol requires a combination of the specialized movers and traditional ROSETTA movers to properly sample the conformational space of the noncanonical molecule and other interacting molecules. Here we describe protocols for designing peptidomimetics that bind target proteins (with specific examples including the design of an oligooxopiperazine inhibitor to the p53-MDM2 protein interaction).

In the following sections, we discuss examples of each of the five noncanonical backbones (OOPs, peptoids, 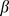-peptides, HBS helices and oligosaccharides) that have recently been added into ROSETTA. The aim of this work is to provide a complete, and thus reproducible, description of all required modifications.

### Oligooxopiperazines

#### OOPs step 1: Determine Residue Subunit

OOPs are helical mimetics with a backbone very similar to that of a peptide but with the addition of an ethylene bridge forming a cycle between adjacent residues (R1 and R2 in figure 1A). To implement a new backbone in ROSETTA, the bonds between atoms must be described in such a way to be compatible with ROSETTA's atom tree representation where explicit cycles are not allowed between residues. We therefore chose a “cut-point” through the ethylene bridge making the OOP residue subunit a single amino acid plus the additional carbon atom. The additional atoms are named either CYP for the first residue of the ring (R1) or CZP for the second residue (R2). The covalent bond between the carbon atoms of neighboring residues, CYP and CZP, is not represented explicitly within the ROSETTA atom tree but rather with a inter-residue connection (described next in step 2, ADD_CONNECT). The inter-residue connection notifies the score function that these two atoms are covalently bonded; this prevents, for example, the close distances between CYP and CZP from incurring a clash penalty.

#### OOPs step 2: Build Patch Files

**Figure 3:**
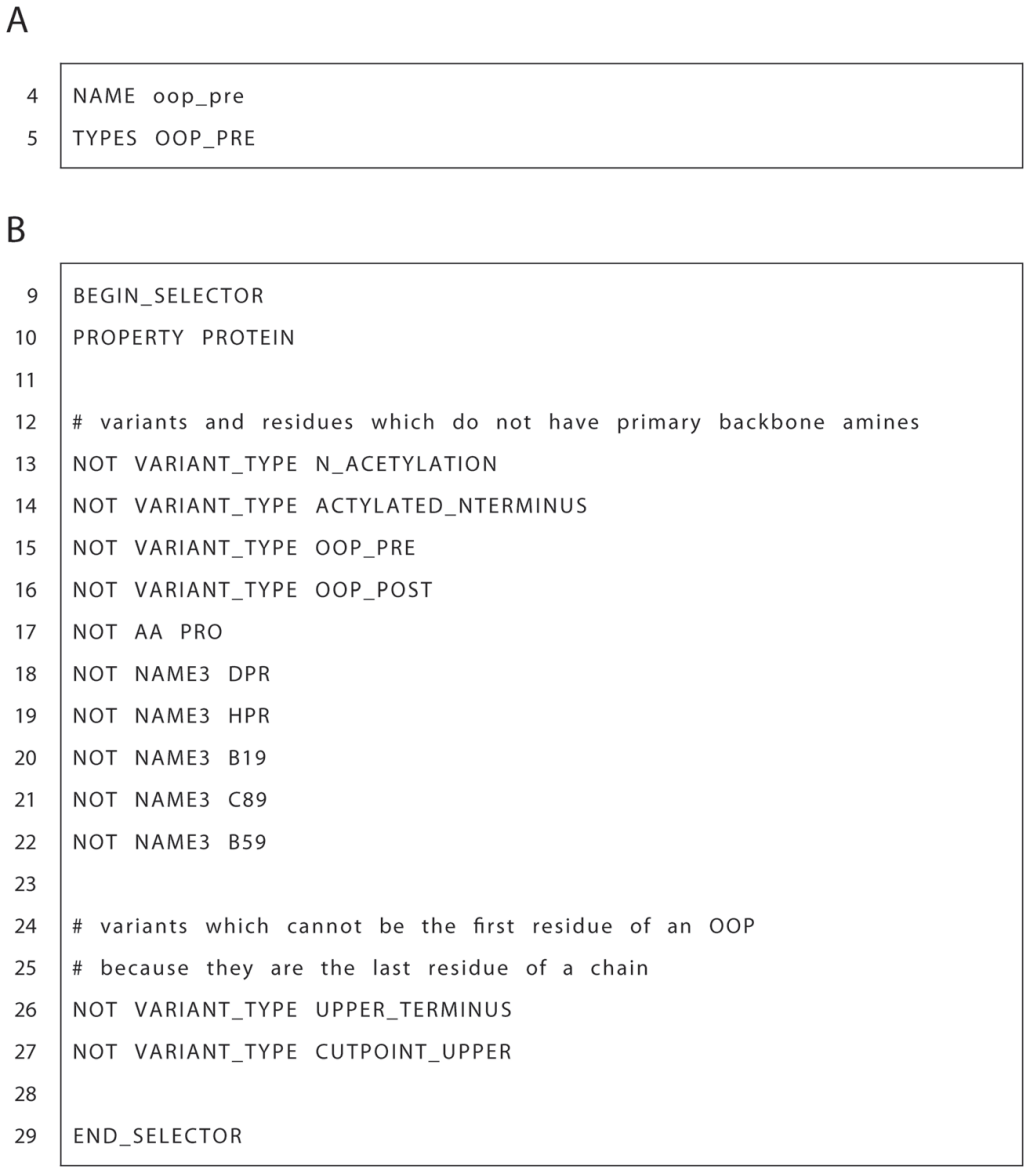
OOP patch name and selector section. A. Sample name and type section of OOP Pre patch file. B. Sample selector section of OOP Pre patch file which describes which ROSETTA ResidueTypes are valid for this patch. Full OOP Pre patch file can be found in file S1.

**Figure 4 pone-0067051-g004:**
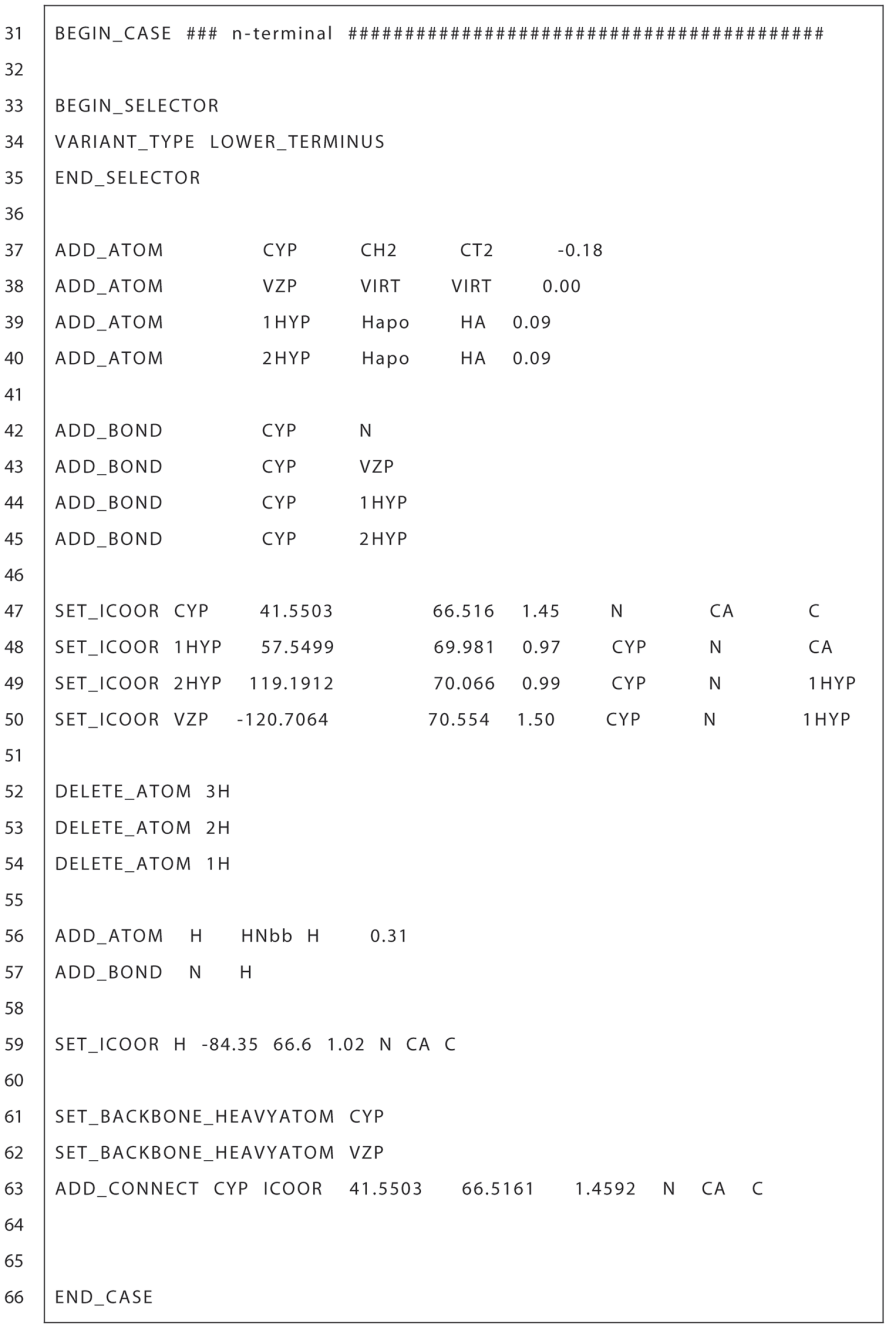
OOP patch N-terminal section. Sample section of the N-terminal section of the OOP Pre patch file. Describes new atoms, bonds and internal coordinates as well as other patch specific parameter declarations. Full OOP Pre patch file can be found in file S1.

OOP scaffolds can be synthesized from most amino acids given that they have a backbone primary amine. We chose ROSETTA's patch system to define ResidueTypes because we do not have to define separate OOP ResidueTypes for all residues defined in ROSETTA (20 canonical in addition to 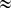
100 noncanonical amino acids). Additionally, it is extensible as all new ResidueTypes added in the future will also have OOP ResidueType variants automatically. We created two OOP patches describing the first (R1) and second (R2) residues of the OOP ring, named the oop_pre patch and oop_post patch respectively. The oop_pre patch file as shown in figure 3, figure 4 and completely in file S1, begins with the declaration of a unique name (NAME) and a variant type (TYPES) (see figure 3A). The VariantType is a class of variants that can be referred to later within ROSETTA to apply specific functionality only to ResidueTypes of that variant class. An example of a more general VariantType is the LOWER_TERMINUS that applies to the collection of all N-terminal residues.

The chemical synthesis of OOPs is incompatible with certain ResidueTypes and therefore we must specify eligible and ineligible residues. The selector section (BEGIN_SELECTOR, END_SELECTOR, figure 3B), declares what ResidueTypes will have the OOP patches applied. The first requirement for an OOP patch is that a ResidueType must have the property of a PROTEIN which restricts the patch to only amino acids (line 10 figure 3B). The next part of the selector section requires ResidueTypes to be synthetically compatible with being an OOP residue (lines 13–22 figure 3B). OOP chemical synthesis requires precursor amino acids with a free primary amine. The restriction is done as a negation (e.g. NOT VARIANT_TYPE) where only ResidueTypes that, for example, do not have acetylated N-termini are applicable. It should also be noted that a ResidueType with two OOP_PRE variants or an OOP_PRE and OOP_POST should not exist and therefore is not allowed. The other entries in this subsection do not allow proline or proline-like amino acids. Finally, OOP_PRE residues with C-terminal variants are not allowed because a residue with an OOP_PRE variant must be immediately followed by an OOP_POST variant residue.

It is often the case that different ResidueTypes may need to have the patch applied differently. The remaining sections in the oop_pre patch file, between the BEGIN_CASE and END_CASE keywords, address different cases of the patch application beginning with the most specific. These sections are where the modifications to the base ResidueTypes are described in order to create a new patched ResidueType. The oop_pre patch has two cases, N-terminal and general. The N-terminal case (figure 4) is defined separately because it has additional hydrogens that must be deleted. The first subsection of the N-terminal case is a selector section similar to the one described above, which defines what ResidueTypes this case applies to (e.g., LOWER_TERMINUS variant residues, line 34 [Fig pone-0067051-g004]).

To properly model a patched OOP residue, ROSETTA requires a full description of the atoms and bonds being added to the base residue. The ADD_ATOM and ADD_BOND keywords describe the atoms and their connectivities, respectively. The ADD_ATOM is followed by a unique atom name, a ROSETTA atom type, a ROSETTA molecular mechanics atom type and a partial charge value (lines 37–40 figure 4). The molecular mechanics atom types are based on CHARMM atom types [42] and therefore specific atom types were chosen based on similarity to CHARMM parameters. The oop_pre patch adds four atoms to the base ResidueType; the CYP carbon, two hydrogens bonded to CYP and a virtual atom VZP. VZP is a placeholder atom that optimally has the same X,Y,Z coordinates of the CZP atom in the connecting oop_post residue. The presence of VZP allows us to later calculate torsion dimensions with respect to the CZP. Additionally, it helps keep a physically realistic OOP ring structure by allowing the minimization of the difference between the X,Y,Z coordinates of VZP and CZP. The ADD_BOND keyword is followed by the unique atom names that are covalently bonded in the patched ResidueType (lines 42–45 figure 4).

The next subsection (lines 47–50 figure 4) declares the internal coordinates of each added atom with respect to other previously declared atoms. The SET_ICOOR keyword is followed by a unique atom name, dihedral value, three-atom angle value, bond length and the unique atom names of the relative atoms. For example, the CYP internal coordinates are described relative to the atoms N, CA and C. The dihedral value describes the dihedral between CYP-N-CA-C atoms (41.5° in the example). The three-atom angle value is the degree angle change of CA from the CYP-N vector (i.e. 180° minus the CYP-N-CA angle value). And the bond length describes the length of the CYP-N bond. Atoms that have internal coordinates defined by LOWER (or UPPER) are defined relative to the preceding residue's C-terminal atom (or succeeding residue's N-terminal atom). This allows the ability to make changes to degrees of freedom that span multiple residues (e.g. 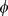 backbone dihedral). It should be noted that these internal coordinate values describe the idealized conformation of the atoms added to the oop_pre residue and the values may change during the course of a protocol. We chose the half-chair conformation coordinates as the oop_pre idealized coordinates because they are the lowest energy according to quantum energy calculations. The actual values are averages of three OOP ring instances in crystallographic data from the Cambridge Structural Database [43] (CSD codes: ZOZTUD, ZARZOH, FOBFEH) and one quantum optimized OOP ring structure.

Lines 52–59 (figure 4) in the example OOP patch file handle the special case of the N-terminus where the three hydrogens bound to the terminal nitrogen are deleted with the DELETE_ATOM keyword and replaced with a single hydrogen. The new hydrogen atom is declared similarly as above.

The final section for the N-terminal case declares the CYP and VZP atoms as backbone heavy atoms (lines 61–62 figure 4) as the ROSETTA movers may distinguish between backbone and side chain atoms. For instance, it is possible to minimize only the side chain atoms of a residue and therefore the CYP and VZP atoms would not be included in the minimization. The last declaration is using the ADD_CONNECT keyword (line 63) that defines the CYP atom as having a covalent bond with an atom in another residue, which will be the CZP atom in the succeeding oop_post residue.

In the oop_pre patch file, the general case is very similar to the N-terminal case just described. Additionally, the oop_post patch file is similar in defining the CZP atom, the virtual atom VYP, and corresponding hydrogens, which make up the oop_post variant ResidueType.

#### OOPs step 3: Build or Obtain Rotamer Libraries

At this stage of implementation it is important to decide whether rotamer libraries need to be built for the new ResidueTypes. The chemistry of OOP side chains is similar to the side chain on their respective amino acids; therefore, we decided that rotamer libraries of the standard ResidueType were sufficient. For instance, a phenylalanine side chain branching off an OOP ring has similar rotamers to that of a typical phenylalanine. Rotamer libraries of the standard ResidueType are the default and no additional declarations are necessary.

#### OOPs step 4: Implement Special Movers

**Figure 5:**
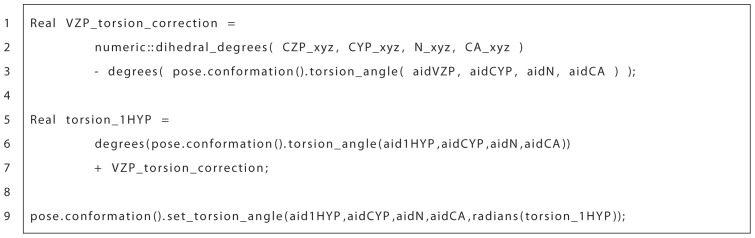
Code snippet for correctly placing hydrogens on the OOP ring. After conformational changes of an OOP ring, hydrogens are often not in ideal positions. This code calculates a correction factor by determining the angle by which a virtual atom and a carbon atom across a cut point align. The torsion angle that defines the hydrogens movement is altered by this correction factor to properly align the hydrogens. A visual representation can be seen in movie S1.

The OOP backbone differs from canonical peptides by the addition of several atoms that form a ring between two residues. We therefore developed additional movers to properly sample the conformational space of the OOP ring, which ROSETTA's peptide-centric movers do not sample properly. The OOP ring has two low energy conformations: a half-chair pucker and a boat pucker, as well as small pucker changes dependent on side chain 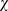 angles. Therefore we created the OopMover class within ROSETTA to change the pucker of an OOP ring. OOP ring movement is determined by the 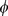 and 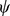 dihedrals of an oop_pre residue (R1). The OopMover class implements functions that set 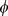 and 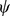 dihedral values of a oop_pre residue and updates the OOP specific hydrogen positions. As seen in movie S1, when the 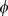 of the oop_pre residue is changed, the VZP virtual atom of the oop_pre residue no longer overlaps the CZP atom on the oop_post residue (again, the VZP atom is a placeholder atom that is meant to track the CZP atom). The difference between the torsion angle of the CA-N-CYP-CZP and CA-N-CYP-VZP is calculated (line 1–3 in figure 5) and the hydrogens (i.e. CA-N-CYP-1HYP torsion) are corrected by this value (line 5–7, 9 in figure 5). The VZP and 2HYP atom positions are corrected automatically because they were defined relative to the 1HYP atom in the oop_pre patch file. A similar method is used for correcting the hydrogens bound to the CZP atom in the oop_post residue, also shown in movie S1.

The OopMover class provides basic functionality to alter the conformation of an OOP ring. More sophisticated OOP classes that rely and extend the OopMover class include OopPuckMover and OopRandomSmallMover that extend the OopMover class. The OopPuckMover makes changes to 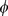 and 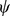 angles in order to change the OOP ring pucker from half-chair (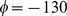, 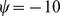) to boat (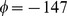, 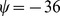) (or vice versa). OopRandomSmallMover randomly changes the 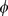 and 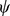 angles of the oop_pre residue by small degree changes to optimize the energy of the ring.

In a final piece of code, we address the need for a constraint on the atoms at the OOP “cut-point”. These atoms, CYP and CZP, are connected insofar that the energy functions treat them as covalently bound. However, a ROSETTA protocol may sample a low energy conformation where CYP and CZP are at a distance outside the range of a covalent carbon carbon bond. Therefore, we implemented the add_oop_constraint function (OopPatcher.cc), where a ROSETTA AtomPairConstraint is placed on the CYP and CZP atoms to ensure they stay within 1.5 Å.

#### OOPs step 5: Create OOPs specific Rosetta Protocol

Once the special movers for the noncanonical backbone have been implemented, the movers can be combined to create a protocol that samples the conformational space of the molecular system of interest. Here we describe a protocol to design an OOP to bind a target protein. Briefly, the protocol iterates between finding low energy conformations and designing low energy sequences on the OOP scaffold while in contact with a target protein interaction interface. The conformational search involves random selection of four movers, 1) rigid body rotation and translation of the OOP scaffold with respect to the target protein, 2) OopPuckMover applied to any OOP rings, 3) OopRandomSmallMover applied to any OOP rings and 4) small angle perturbations to the 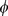 and 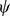 torsion angles between OOP rings. This conformational search via randomly selected movers iterates 100 times. The protocol then applies a design phase (repack) where side chains on the OOP scaffold are substituted for lower energy ones. Side chains on both sides of the interface are repacked to find low energy combinations of rotamers.

The protocol performs 10 cycles of the conformational search followed by the design phase. After a thousand independent runs of the total protocol are complete, the top five percent of final poses in terms of ROSETTA total energy are filtered and then sorted by the binding energy between the OOP scaffold and the target protein. One should manually inspect final designs for good packing and proper interface inhibition. To make the protocol widely available, we have also included the protocol in the ROSIE server (**NCBB Design**, http://rosie. rosettacommons.org). Users can upload a protein target with an OOP scaffold near the inhibition interface (PDB format) and run the protocol which designs an OOP to recognize a target interface.

### Peptoids

#### Peptoids step 1: Determine Residue Subunit

Peptoids (N-substituted glycine oligomer units) and peptides have a identical backbones but unlike OOPs, they do not have a cycle in the backbone between neighboring residues. This simplifies determining the residue subunit and we therefore chose a residue subunit for peptoids to be the same as for peptides (figure 1B). Having an identical repeating unit additionally allows peptides and peptoids to be swapped easily within protocols allowing for the creation of peptide/peptoid hybrid molecules.

#### Peptoids step 2: Build Parameter Files

Peptoids are commonly produced using a submonomer synthesis which has allowed more than 200 different primary amines to serve as peptoid side chains [44]. This is important because even though peptoids and peptides have an identical backbone structures, their side chains can be quite different. In light of this, peptoid ResidueTypes were implemented using the params system, which allows a user to make residues that are not derived through modification of other residues.

**Figure 6:**
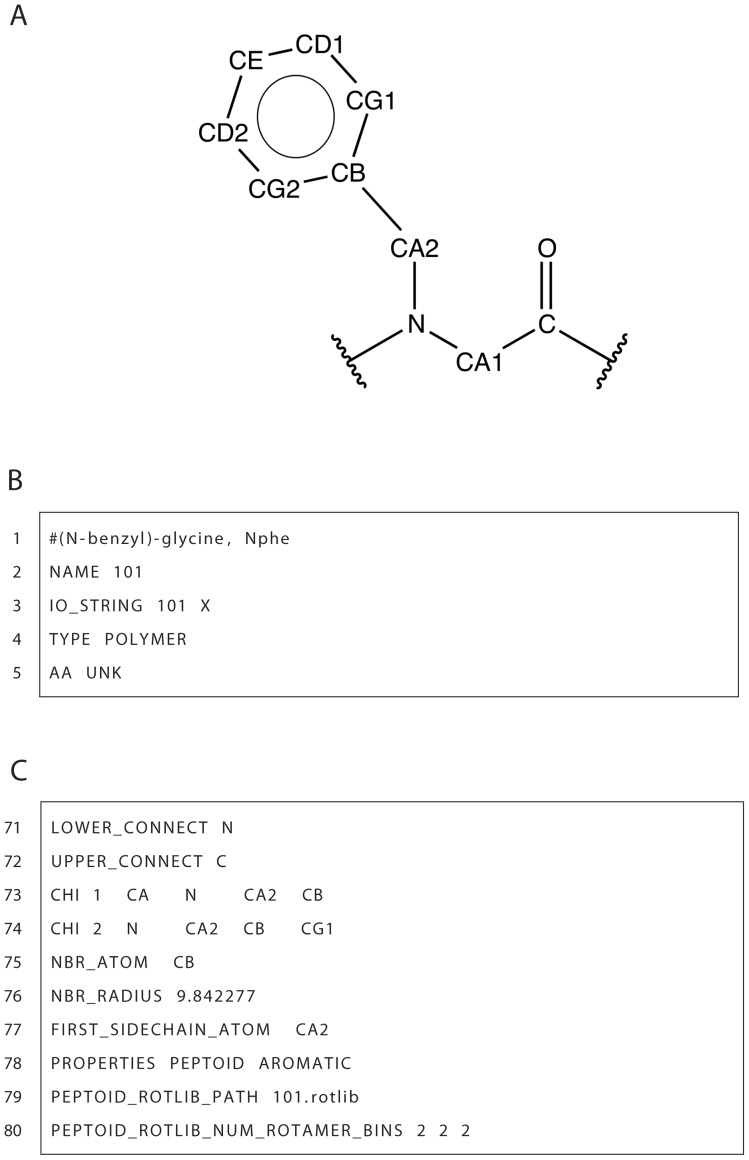
Chemical structure of peptoid NPhe and ResidueType parameter file. A. The schema shows the atom names and bond connectivity of the example peptoid residue type. B. Name and type section of NPhe parameter file. C. ResidueType specific definitions for Nphe parameter file. The full NPhe ResidueType parameter file can be found in file S2.

In figure 6A, we show an example peptoid ResidueType (*N*-benzyl)-glycine (i.e. NPhe). The NPhe parameter file, shown in part in figure 6B–C and completely in file S2, defines a unique name with the NAME keyword and three-letter and one-letter codes with the IO_STRING keyword. In this example, the name and three-letter code are identical (although not a requirement). All NCAAs use the one-letter code of “X” due to the inability to represent all peptoid residues with a single letter. Line 4 (figure 6B) defines this peptoid ResidueType as a polymer and line 5 (figure 6B) sets the property AA to be unknown (UNK) which is used for all NCAAs. The AA property is used by portions of ROSETTA code dealing with knowledge based energy terms (among other functions) and since these energy terms are not trained on peptoid structural data, the code needs to be notified of its noncanonical nature. Lines 7–46 (file S2) are very similar to the patch files described above. The ATOM and BOND keywords define the atoms and bond connectivity for the entire residue. Lines 48–69 (file S2) define the internal coordinates of each atom with respect to other defined atoms. The format is the same as described above for the OOP patch SET_ICOOR definitions.

As noted above in the OOP section, molecular mechanics atom types are chosen based on CHARMM atom types but unfortunately, there is not a similar CHARMM atom type to a peptoid's tertiary backbone nitrogen. To obtain parameters for this nitrogen, we ran quantum energy calculations on peptoid model structures and adjusted the CHARMM proline nitrogen parameters based on these calculations. This provides a reasonable approximation to the behavior of torsion angles involving the peptoid backbone nitrogen.

Lines 71 and 72 (figure 6C) define the polymeric connection atom for both the N-terminal (LOWER_CONNECT) and the C-terminal (UPPER_CONNECT) and are the atoms that are bonded to neighboring residues. On lines 73 and 74 (figure 6C), the keyword CHI defines the degrees of freedom of side chain torsion angles and is followed by a number indicating the CHI angle index and four atoms that define the torsion. On lines 75 and 76 (figure 6C), the neighbor atom (NBR_ATOM) and radius (NBR_RADIUS) are defined. These parameters are used by ROSETTA scoring terms which depend on the number of neighbors a residue has to determine the residue environment (buried or surface) and speeds calculations of short range two body energies (see Leaver-Fay et al. 2011 ROSETTA3) [41]. Line 77 (figure 6C) defines the first atom of the side chain and line 78 declares additional characteristics (that this is a PEPTOID and has an AROMATIC function group in its side chain) of this ResidueType through the PROPERTIES keyword. ResidueType properties are used in ROSETTA for boolean checks (e.g. if there are peptide and peptoid versions of a kinematics function). Finally, lines 79 and 80 (figure 6C) define the file system path to the rotamer library in the ROSETTA database to be used for this ResidueType and the number of rotamer bins.

Parameter files, similar to the one just described, have been created for over fifty peptoid side chains. Additionally, N-terminus and C-terminus variant ResidueTypes (created using the patch system) and the ability to create cyclic peptoids are available.

#### Peptoids step 3: Build or Obtain Rotamer Libraries

Side chains on peptoids interact differently with the backbone than their peptide analogues [15], resulting in very different 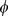, 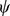, 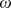 and 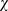 distributions and very different backbone-side chain conformation dependancies. Therefore, new rotamer libraries need to be created to properly sample the degrees of freedom of peptoid side chains. Additionally, 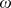 dihedral angles in peptoids readily adopt *cis* and *trans* conformations and rotamers are therefore dependent on the preceding residue's 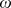 angle. With these dependencies in mind, we built rotamer libraries for all peptoid ResidueTypes using the MakeRotLib protocol [39]. The protocol was modified to sample both *cis* and *trans* for the preceding 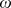 angle to properly account for interactions of side chain atoms and preceding carbonyl oxygens.

#### Peptoids step 4: Implement Special Movers

As with the OOP backbone, peptoids also have a move set separate from canonical peptides. Peptoids therefore need special movers to properly sample their conformational space efficiently. Three movers were added to ROSETTA to sample peptoid conformational space: RandomTorsionMover, RandomOmegaFlipMover and the CyclizationMover. RandomTorsionMover is comparable to the SmallMover used for peptides [45] however in addition to 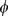 and 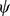 the RandomTorsionMover perturbs the 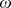 dihedral by adding or subtracting a random number of degrees up to a predefined amount. It is intended for minor adjustments to optimize the current conformation. Unlike SmallMover, RandomTorsionMover is not guided by ROSETTA's Ramachandran backbone torsional potential (due to its dependence on peptide statistics from the Protein Data Bank). RandomOmegaFlipMover is used to switch between *cis* and *trans* 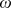 angles and is for large jumps in conformational space. It functions by randomly choosing from a list of allowed positions and adding 180° to the 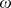 dihedral.

Often functional peptoids are cyclized, covalently linking the N and C-termini, in order to stabilize their structure into a macrocycle. To appropriately model a macrocycle peptoid and allow for optimization of all degrees of freedom within ROSETTA (and its atom tree data structure), a virtual covalent bond is made between the N-terminus atom on the 1st residue and the C-terminus atom on the last residue. The CyclizationMover uses minimization to close cycles (if they have been broken during a perturbation) and updates atom positions on either side of the virtual bond when there are changes to degrees of freedom that are relative to atoms on both sides. This update is done in a similar fashion to the OopMover described above using virtual atoms as placeholders. Although initially developed for peptoid applications, all three movers can function on peptides as well.

#### Peptoids step 5: Create specific Rosetta Protocol

The peptoid design protocol provided here is (like the OOP design protocol) a protocol to design protein-interface antagonists, but contains a modified perturbation phase where the OOP specific movers have been replaced with the RandomTorsionMover and the RandomOmegaFlipMover. The RandomOmegaFlipMover is sampled once for every 100 rigid body rotation, translation or random torsion moves (RandomTorsionMover). Every 100th perturbation step, and immediately before the design phase, the cycle is closed using the CyclizationMover if the peptoid is cyclic. To make the protocol widely available, we have also included the protocol in the ROSIE server (**NCBB Design**, http://rosie.rosettacommons.org).

### 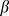-peptides

#### 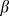-peptides step 1: Determine Residue Subunit

As stated above, the backbone of 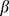-peptide is similar to that of canonical peptides, with one extra backbone carbon atom. Therefore we use the same residue subunits as canonical residues (figure 1C).

#### 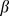-peptides step 2: Build Parameter Files


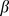
-peptides can be implemented in ROSETTA either through patches of standard protein ResidueTypes (see OOP section above), or through the creation of new ROSETTA ResidueTypes (see peptoid section above). The 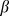-peptide framework was initially developed in the beginning stages of ROSETTA3, when the full patch functionality was not available; therefore the 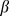-peptide implementation creates new ResidueTypes using the params system. Because the 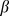-peptide residues have exactly the same side chain atoms as the canonical amino acids, the params files are simply derivatives of the canonical ResidueTypes. We used a python script (i.e. rosetta_tools/beta-peptide/create_beta_peptide_params.py) to generate the params files for all 20 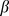-peptide residues from the canonical residue types automatically. The script simply adds an extra carbon atom CM (and the hydrogen atoms attached to it) to the backbone, and adjusts bonding information and ideal coordinates for this new atoms and its bonded neighbors atoms. Currently only 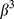-peptide ResidueTypes are implemented. The creation of 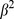-peptide ResidueTypes would consist of a similar process.

#### 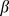-peptides step 3: Build or Obtain Rotamer Libraries

Since 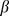-peptides have similar side chains as canonical peptides branching off a backbone carbon atom, the current implementation assumes that they have approximately the same side chain rotamers. Therefore we used the rotamer libraries of canonical residue types for the corresponding 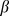-peptide residue types. One caveat of this approach is that these rotamer libraries inherit the same backbone torsional dependence from the corresponding canonical peptide, with 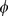 and 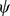 corresponding to the C-N-CA-CM and N-CA-CM-C torsion angles respectively. Because the backbone atoms of the 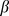-peptide are different from the canonical peptide, a better approach might be to use a MM potential to minimize the side chain rotamers for each backbone configuration as has been done in the MakeRotLib protocol [39]. Additionally, rotamers from [40] could also be used. These improvements are not yet implemented in ROSETTA, but may be important for future design efforts.

#### 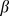-peptides step 4: Implement Special Movers

The current 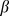-peptide designing protocol performs fixed-backbone side chain design, and uses the standard ROSETTA movers only.

#### 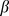-peptides step 5: Create specific Rosetta Protocol

With the new parameter files, we created a protocol for fixed-backbone design and sequence replacement of 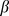-peptides. Briefly, the protocol fixes the backbone of the input model and searches for the lowest-energy combination of the side chains for residues of interest (as specified by user). The protocol then outputs the lowest-energy model, which has the best combination of the side chain identities and rotamers sampled. This protocol was applied to redesign the core residues of an octameric 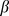-peptide bundle [46]. For the design of the 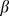-peptide bundle, we also include the functionality for symmetric design, where we can force equivalent residues to have the same side chain identities and rotamers. To make the protocol widely available, we have also included the protocol in the ROSIE server (**Beta_peptide_design**, http://rosie.rosettacommons.org).

### Hydrogen Bond Surrogate (HBS) helices

#### HBS step 1: Determine Residue Subunit

The HBS scaffold constrains a peptide to an 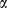-helical conformation by converting the hydrogen bond between the *i* and *i*+4 residues of an 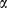-helix to a covalent connection. We determine the first residue subunit of an HBS macrocycle to be a combination of the *i*, *i*+1 and *i*+2 residues of an 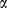-helix (see figure 1D). The conversion from a hydrogen bond to a covalent bond forms a cycle which cannot be explicitly modeled by ROSETTA's internal atom tree representation. Similar to the OOP implementation described above, the HBS scaffold requires a “cut-point” where the first residue in the HBS cycle has 5 additional carbon atoms (plus one additional oxygen) and the third residue has 1 additional carbon.

#### HBS step 2: Build Patch Files

Similar to the OOP implementation, we chose the patch system for building new HBS ResidueTypes. This requires two patches, hbs_pre and hbs_post to describe the new atoms that should be applied to the first and third residues of the HBS macrocycle, respectively. One feature unique to the HBS helix is its long linker. Since the linker has a larger radius than most residue side chains, the neighbor radius needs to be adjusted to properly determine the nearby residues in three-dimensional space (residues that are close to the linker but not the side chain). Because of this size increase, the NBR_RADIUS is extended to 8.0 Å from the CA atom for all variants of hbs_pre.

#### HBS step 3: Build or Obtain Rotamer Libraries

In the case of HBS backbones, the new ResidueTypes are derivatives of typical ResidueTypes, and therefore side chain rotamers are expected to be similar. We thus use the rotamer libraries from the base ResidueType.

#### HBS step 4: Implement Special Movers

As there is a “cut-point” introduced in the HBS linker, a ROSETTA AtomPairConstraint is necessary to keep the distance fixed between the two atoms on either side. We implemented functionality similar to the add_oop_constraint to automatically detect HBS patched residues and apply this constraint. Additionally, the HBS linker is expected to have a fairly stable conformation according to NMR studies [28]. Because of this conformational stability, no special movers are currently implemented to sample linker conformations. If one chooses, however, a mover similar to the OopMover described above may be useful to get more fine grained moves if further optimization of the linker is necessary.

#### HBS step 5: Create Rosetta Protocol

We have created a protocol to design potential high affinity HBS binders to a given target protein. The protocol iterates between rigid body perturbations and design of user specified residues (unspecified residues are repacked) to find a low energy conformation. To make the protocol widely available, we have also included the protocol in the ROSIE server (**NCBB Design**, http://rosie.rosettacommons.org).

### Oligosaccharides

#### Oligosaccharides step 1: Determine Residue Subunit

The “backbone” of a oligosaccharide is a chain of rings – composed of carbons closed by an oxygen atom – in addition to exocyclic atoms connecting those rings. Since the oligosaccharide backbone differs significantly from a peptide backbone, we could not modify the standard peptide subunit to create the saccharide subunit, as was done in prior examples. Instead we defined each ring as a separate subunit where the start atom of the residue is the anomeric carbon (labeled 1 in figure 1E) and the last atom is the oxygen in the glycosidic bond. (The full subunit is shown in brackets in figure 1E). As mentioned above for the case of OOPs, explicit cycles are not compatible with ROSETTA's atom tree. We chose to add a cut-point between the cyclic oxygen atom and the anomeric carbon, because this is the bond formed when a linear saccharide isomerizes to its common cyclic form.

#### Oligosaccharides step 2: Build Parameter and Patch Files

The number of possible saccharide ResidueTypes is quite large due to the variability of ring size (five- or six-membered rings), number of carbons (between three and nine), position of glycosidic bond connections, stereoisomerism of the carbons, and the diverse set of side chain groups that can be attached to most of the carbons in the ring. To deal with this complexity, our strategy has been to use a combination of the params system and the patch system. The “base” saccharides – single rings with only hydroxyl groups attached to the ring carbons – each have their own parameter file. This requires a separate parameter file for each ring size, glycosidic bond connection, and stereoisomer combination. Patch files are then used for applying any conceivable side chain to the base saccharide ResidueTypes.

#### Oligosaccharides step 3: Build or Obtain Rotamer Libraries

The “base” saccharides, which only have hydroxyl groups as side chains, do not require rotamer libraries. To properly orient the hydroxyl hydrogen atom, a set of PROTON_CHI angles (C
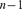
–C
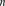
–O
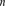
–H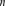) is defined in the parameter file for each hydroxyl group. The PROTON_CHI keyword is followed by the torsion ID and a listing of three torsion angle samples of 60, −60°, and 180°.

For modified saccharides, those with non-hydroxyl side chains, we define rotamers by identifying low-energy side chain conformations. In the absence of experimental structures from which to derive statistical potentials, we scanned the side chain chi angles and ran quantum mechanical calculations on the resulting conformations (Gaussian software package [47] at a HF/6-31G(d)//MP2/6-31G(d) level of theory). Energy plots were created and energy wells within 1 kcal/mol of the lowest energy were selected by visual inspection as rotamer bins. Once rotamer bins were selected, the ADD_CHI_ROTAMER keyword was used in the patch file to specify backbone independent rotamers.

#### Oligosaccharides step 4: Implement Special Movers

Oligosaccharide backbones are not compatible with the peptide centric movers in ROSETTA and therefore require a unique set of movers to alter backbone torsion angles and ring conformations. Oligosaccharides have rotatable torsion angles about their glycosidic bonds defined as 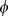 and 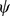 as shown in figure 1E. Additionally, 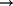
6 linked oligosaccharides also contain a torsion angle defined as 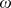. Since this nomenclature overlaps that used for peptides, we modified the ROSETTA code for getting and setting 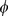, 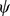 and 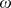 (Pose.cc). The code recognizes whether a peptide or saccharide residue is being modified and accesses the proper torsion angles in the AtomTree. This allows the use of standard ROSETTA movers such as small and shear moves. A RingConformationMover has also been developed, similar to the OopPuckMover described above, to use internal ring torsion angles to sample various ring conformations.

#### Oligosaccharides step 5: Create Rosetta Protocol

We developed a docking protocol for the prediction of the structure of oligosaccharide–protein complexes. The protocol starts with a pseudo-random perturbation of the oligosaccharide backbone 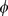/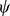 angles biased towards energy minima obtained from pre-computed 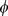/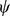 energy maps from quantum calculations for specific saccharide residue pairs (the same quantum parameters were used for rotamer libraries described above). The standard ROSETTA small and shear movers are then used in conjunction with the RingConformationMover to account for sugar backbone flexibility. Additionally, the standard RotamerTrialsMover is used for sampling saccharide “side chains”. To maximize the conformational search space, the sequence of moves is repeated multiple times while simultaneously ramping the van der Waals repulsive and attractive terms in the score-function up and down respectively. In the final step, the rigid body orientation of the oligosaccharide–protein complex and the residue side chains are simultaneously minimized. We successfully tested the protocol by docking a heparin-like six-residue oligosaccharide to protein BT4661 as a part of the 2012 Critical Assessment of the PRediction of Interactions (CAPRI) challenge, resulting in one acceptable prediction.

### Noncanonical Backbones and compatibility with peptide-centric Rosetta movers and protocols

Due to the peptide-centric nature of ROSETTA, many of the movers and protocols were developed with only peptides and proteins in mind. Many general movers in ROSETTA (such as packing, rigid body perturbations and minimization) however still apply to noncanonical backbones and can be used seamlessly. Additionally, protocols built upon these general movers can be used with noncanonical backbones with minor adjustments to the commandline. For instance, to use the FastRelax protocol, which is based on packing and minimization, one needs to load any required ResidueTypes not on by default with a commandline option (e.g. -include_patches patches/oop_pre.txt patches/oop_post.txt) or uncomment appropriate lines in the residue_types.txt or patches.txt found in the ROSETTA database. Additionally, since compiled protocols may not call specific constraint setup functions such as the AtomPairConstraint function described above, constraints should be defined on the commandline using the ROSETTA constraint file interface.

Other ROSETTA movers, however, assume canonical peptide atom names and connectivity or were created specifically for peptide movement. These may produce undefined behavior when applied to noncanonical backbones. For example, the commonly used SmallMover is designed to alter a peptide's 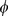 and 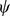 backbone torsion angles. In ROSETTA, 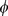 and 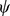 are defined as the first and second torsions respectively found along the backbone starting at the LOWER_CONNECT atom and ending at the UPPER_CONNECT atom. If a specific noncanonical backbone has redefined or has additional backbone torsion angles, the SmallMover may not be appropriate for use because it may alter unexpected torsions or will not sample all torsions necessary for complete sampling. An illustrative example of how to make noncanonical backbones compatible with existing movers can be found in the oligosaccharide section where it was made compatible with the SmallMover. In all cases, however, we advise careful consideration of all movers used in noncanonical backbone protocols.

## Results

Implementation of noncanonical backbones is enabling a number of novel applications in peptidomimetic structure modeling and design. Examples, including detailed experimental tests, have been published recently or are in preparation for publication in application-specific manuscripts. As the primary purpose of this manuscript is to explain the conceptual implementation of noncanonical backbones in ROSETTA, we concentrate below on results that illustrate the steps described in the Methods section, with specific examples from score function comparisons and design of a protein-protein interaction inhibitor with oligooxopiperazines.

### Score function validation using quantum calculations

The original ROSETTA score function was trained on protein structures from the Protein Data Bank (PDB). This knowledge-based energy function is useful for certain applications of noncanonical backbones (i.e. fixed backbone) but does not apply to applications with large backbone sampling. One reason why is that statistics from the PDB may not be readily transferred to the behavior of noncanonical backbones. For example, relationships between 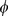, 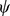 and 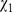 angles do not have any correspondence between peptoids and peptides. A second reason why a knowledge based score function is insufficient is that there are not enough samples of experimentally solved structures of the specific noncanonical backbones to properly retrain a new statistics-based scoring function. Fortunately, ROSETTA has recently been extended to include a physics-based molecular mechanics score function, mm_std, developed by Renfrew et al. [39] that does not rely on knowledge-based terms but incorporates terms from the CHARMM force field [42] in combination with ROSETTA full-atom scoring function.

In this section, we compare the ROSETTA mm_std score function to a more accurate, albeit more computationally intense, quantum (QM) energy calculation. The comparison analysis of different OOP conformations provides a performance measure to determine the accuracy of the ROSETTA energy function using noncanonical backbones as well as a roadmap for future noncanonical backbone implementations to follow in order to ensure the accuracy of energy calculations. The QM calculation was carried out using the Gaussian software package [47] with a Hartree-Fock optimization followed by a B3LYP energy calculation and a 6–31G(d) basis set. Energy comparisons were made for 1) backbone 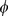 and 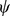 dihedral angles between two OOP rings, and 2) the side chain 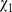 angle for both half-chair and boat OOP ring conformations.

**Figure 7:**
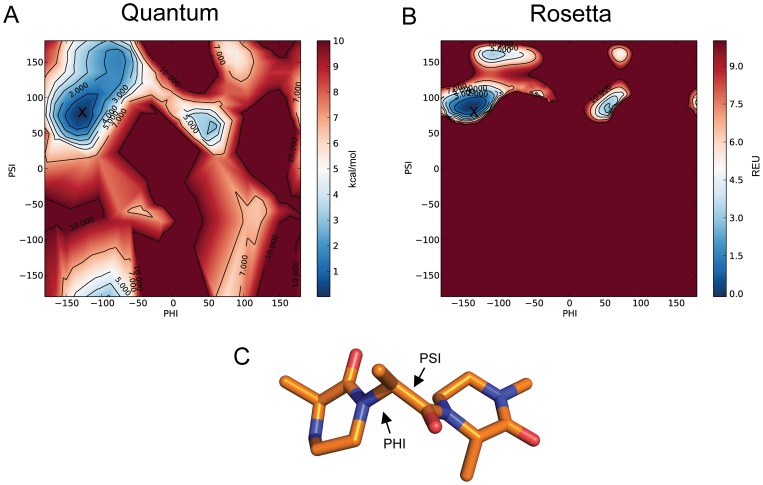
Validation of mm_std score function on OOP dimer. Noncanonical backbones often require score functions that are based on molecular mechanics rather than the traditional ROSETTA knowledge based terms. A) Quantum mechanics (QM) and B) ROSETTA mm_std energy calculations of 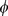 and 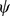 torsion angle combinations for an OOP dimer (C). The 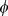 and 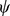 torsion angles are of the linker residue between two OOP rings and are labeled in C. Blue regions represent low energy (high probability) conformations, red regions represent high energy (low probability) conformations. The QM plot is measured in Kcals/mol while the mm_std plot is measured in ROSETTA Energy Units (REU). The ROSETTA mm_std calculations recover the main low energy wells as predicted by QM calculations with the lowest energy conformation estimated by QM marked by an ‘X’ on both plots. The structure in (C) is of the low energy conformation as predicted by QM.

Figure 7 shows a Ramachandran plot of the 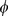 (x-axis) and 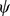 (y-axis) dihedral angles between two OOP rings where regions colored in red are high energy and those colored in blue are low energy. Figure 7A, shows a plot based on the quantum calculations. The low energy wells are fewer and smaller than a peptide Ramachandran plot and therefore suggests the OOP is more stable. This is expected due to the steric hindrance between the additional atoms in the OOP rings.

Figure 7B shows the Ramachandran plot based on the ROSETTA mm_std score function. The ROSETTA mm_std score function captures the low energy wells of the quantum results. Additionally, the lowest energy conformation from the quantum optimizations, marked by an ‘X’, is in a low energy well in both plots. There are additional regions of the QM plot that are not captured by the mm_std score function. This is most likely due to the ability of QM optimization to alter bond lengths and bond angles which is not done in ROSETTA optimizations. This result does, however, suggest that the ROSETTA mm_std score function is a good approximation to the likely energy landscape and can be used to accurately estimate energies of conformations with varying backbone dihedral angles.

**Figure 8:**
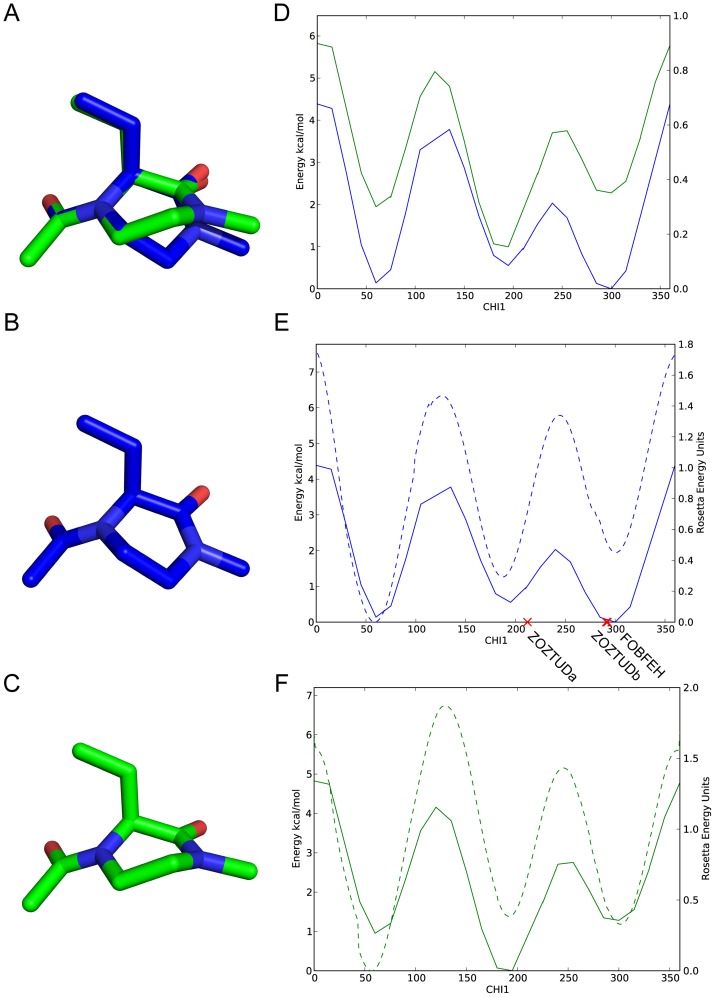
OOP ring conformations and validation of mm_std score function on side chain dihedrals in different ring conformations. A) Overlay of half-chair (blue) and boat (green) OOP ring conformations. B) Structure of half-chair OOP ring conformation. C) Structure of boat OOP ring conformation. D) QM energy calculations (Kcal/mol) of different side chain 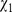 dihedral angles. Blue represents half-chair conformation. Green represents boat conformation. The half-chair conformation is lower in energy in two out of the three 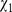 dihedral energy wells (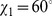 and 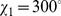) but nearly isoenergetic with the boat conformation in the third (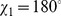). E) QM energy (solid) and ROSETTA mm_std (dash, ROSETTA Energy Units) energy calculations for 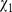 dihedral angles with OOP ring in half-chair conformation. Red X's show 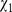 values of oxopiperazine side chains (labeled with Cambridge Structural Database code). F) QM energy (solid) and ROSETTA mm_std (dash, ROSETTA Energy Units) energy calculations for 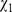 dihedral angles with OOP ring in boat conformation. For both (E) and (F), ROSETTA mm_std score function recapitulates the low energy minima.

It is also important to test the performance of side chain dihedrals when implementing a noncanonical backbone. Side chain groups branching off OOP rings may encounter steric effects from the additional atoms in the OOP ring. The OOP ring can be in either a half-chair (figure 8B) or boat conformation (figure 8C) (overlay is shown in figure 8A) and the energy of the side chain 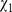 angle is affected by this ring puckering. Figure 8D shows an angle vs energy plot of the quantum energy calculations similar to the parameters described above. The half-chair (blue) is consistently lower in energy across all 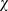 angles sampled than the boat conformation (green) but the rotamer at 180° is nearly isoenergetic between the half-chair and boat conformations. This reinforces the importance of the OopPuckMover conformational sampling class described above, as an OOP residue may occupy both conformations with similar probability.

ROSETTA mm_std score function calculations were also computed (dashed lines) and overlay the quantum results (solid lines) for both the half-chair (figure 8E) and boat (figure 8F) conformations. These comparisons show the ROSETTA mm_std score function accurately reflects the energy behavior of the 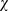 angle by properly aligning minima in each of the quantum low energy rotamer wells. Additionally, there are two crystal structures of oxopiperazine half-chair rings with side chains in the Cambridge Structural Database [43], one with a tyrosine side chain (code: FOBFEH) and another with two phenylalanine side chains (code: ZOZTUD). The 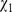 values of these side chains are marked as red X's in figure 8E and show correspondence with the minima of the energy landscape. This side chain-scanning result and the backbone-scanning result above suggest ROSETTA is capable of accurately calculating energies of a general class of noncanonical backbone conformations.

### Noncanonical backbone applications on the ROSIE server: a design example

In an effort to increase the usability of ROSETTA molecular modeling suite, ROSETTA applications can now be installed in a unified webserver framework called ROSIE (http://rosie.rosettacommons.org), which provides a user friendly interface to ROSETTA without the traditional need of Unix development skills. Server applications using several of the noncanonical backbones described above have been implemented into the ROSIE (ROSETTA Online Server that Includes Everyone) framework and are available for public use. Here we describe results from the **NCBB Design** server application where we design an OOP dimer to target MDM2 and inhibit the MDM2-p53 protein interaction.

**Figure 9:**
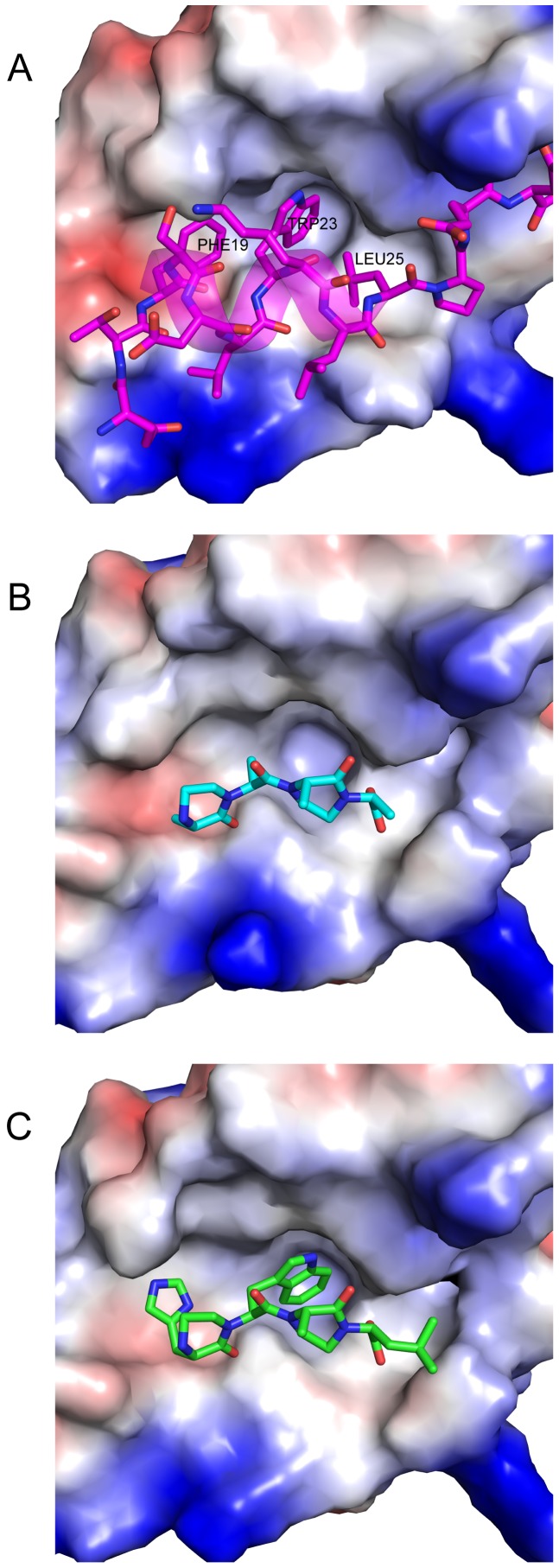
Demonstration of the NCBB Design Server Application. The OOP scaffold was designed to inhibit the p53-MDM2 protein interaction using the **NCBB Design** server. A) Crystal structure of p53 (pink sticks) - MDM2 (electrostatic surface) protein interaction with three hotspot residues highlighted (Phe19, Trp23, Leu26) which are responsible for majority of interaction's binding affinity (pdbid: 1YCR). B) Starting structure of alanine OOP scaffold (cyan) placed into the binding pocket of MDM2. C) Final designed structure reported by **NCBB Design** server. The final design shows a histidine designed in the first position recovering an aromatic residue similar to the hotspot Phe19. A tryptophan is designed in the second position recovering the hotspot residue of Trp23 and a Leu is designed in the fourth position recovering the hotspot residue Leu26.

p53 is a tumor suppressor protein that becomes activated when cells encounters genomic instability or cellular stress [48], [49]. When activated, p53 enters the nucleus and acts as a transcription factor to turn on genes related to apoptosis and cell cycle arrest. MDM2, an E3 ubiquitin ligase, is a negative regulator of p53 and when overexpressed, depletes p53 from the cell, which often leads to cancer [50], [51]. Inhibition of the p53-MDM2 protein interaction has been the focus of several efforts to develop small molecule inhibitors that antagonize this interaction [52], [53]. A high resolution crystal structure of the MDM2-p53 protein interaction is available in the PDB (pdbid: 1YCR)[54] and was used to create a starting structure (figure 9A). There are three residues on p53 Phe19, Trp23 and Leu26 that are deemed hotspot residues and are responsible for the majority of interaction's binding affinity. The three residues are at the *i*, *i*+4 and *i*+7 positions of an 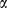-helix on p53 and make substantial hydrophobic contact with MDM2. Since OOP analogs can mimic these positions on an 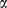-helix, we aligned an OOP dimer with four alanine residues onto these hotspot residues to provide a starting conformation (figure 9B). This strategy of manually placing a noncanonical backbone scaffold onto hotspot residues should provide good starting conformations for most targets where hotspot residues are known from experimental evidence or predicted using computational methods such as ROSETTA alanine scanning [7], [55]. The starting structure is therefore made up of two chains, MDM2 and the OOP scaffold, and is in PDB format.

Next, the starting structure was uploaded using the **NCBB Design** submission form. The server runs the oop_design application which iterates between a perturbation phase and a design phase for a default of 10 cycles. The perturbation phase consists of a random 100 perturbations (default) selected from rigid body moves of the OOP with respect to the target protein, small OOP moves, OOP pucker moves and small angle moves to inter-OOP ring backbone torsion angles (residues patched with oop_post are not constrained and therefore behave similarly to peptides). The design phase consists of side chain substitutions at the specified OOP residue positions (1, 2 and 4, selected on the job submission form) and repacking of side chains at all other positions. This is followed by a minimization step and a return to the perturbation phase. After 1000 decoys (independent runs) are produced, the top 5% based on total score are filtered. These top decoys are then sorted by binding energy and a link to the top one is provided to the user. Additionally, a link to the full set of decoys and scores are provided for user inspection.

Figure 9C shows a final model returned by the server. The final designed OOP has a sequence of HWAL and as seen in figure 9C, the Trp in the 2nd position on the OOP fills the same pocket as the p53 hotspot Trp23 in the crystal structure (figure 9A). The p53 Phe19 hotspot pocket is filled in the top design with a similar residue, a His, which suggests the pocket is flexible enough to accommodate aromatic residues. Finally, a Leu is designed in the fourth position of the OOP scaffold but in a different orientation than the hotspot Leu26 on p53. A possible reason for this maybe the starting conformation of the OOP scaffold with respect to the target protein. Creating several starting conformations and running them through the server is recommended to diversify sampling.

Once a final design is produced, it can be synthesized and experimentally validated as an inhibitor of the p53-MDM2 protein interaction. Several OOP inhibitor designs created by this computational method targeting the p53-MDM2 interaction have been synthesized and experimentally validated. ROSETTA designs show an improvement of binding from micromolar 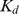 (OOP inhibitors designed by non computational methods) to nanomolar 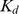 (unpublished data). This improvement of binding suggests that computational design using noncanonical backbones in ROSETTA is a powerful approach to discovering high affinity inhibitors to protein-protein interactions.

## Discussion

Protein interactions are an essential part of biological function. Intracellular events including cellular signaling, transcription, and the cell cycle are regulated by protein interactions. Some current estimates of the number of protein interactions in the Human interaction network are greater than 300,000 [56]. Of these estimated interactions, nearly 15,000 protein complexes are in the Protein Data Bank [57], the majority of which have well defined secondary structure at a well packed portion of the interface [7]. Unfortunately, most small molecule pharmaceutical searches of druggable targets have been limited to proteins with small, well defined pockets (e.g. enzymes and receptors) while protein-protein interactions have been considered difficult targets because of their larger and relatively flat interfaces [58]. There is great interest in therapeutic antibodies because they are capable of targeting protein interactions [59] but unfortunately antibodies have poor bioavailability and cell permeable properties [58]. These factors limit their use as therapeutics.

Peptidomimetic based inhibitors can address these limitations because they can be synthesized to be larger than small molecule drugs. Additionally, peptidomimetics are generally more stable and proteolysis-resistant than the natural secondary structures they mimic [60]. The implementation of peptidomimetics into the ROSETTA framework is a first step in providing the computational infrastructure to efficiently sample the space of possible functional groups that will increase binding to a given target of therapeutic interest.

Recently, CHAMP [61], a computational design method, has been extended to incorporate 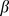-peptides [62]. The method was successful in designing a 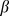-peptide targeting the TM helix of an integrin. The CHAMP method however is limited to targeting transmembrane helices. Another computational design method, NAPOLI [63], is capable of designing arbitrary backbones (including 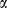, 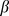 and 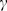-peptides), but its application is mainly focused on the design of helical bundles. By developing a broad modeling framework, we have created the ability to evaluate and design several promising oligomeric systems to target protein interactions.

The backbone implementation protocol we describe here (figure 2) is generally applicable to a diverse set of polymers of interest. This expands the utility of the ROSETTA modeling and design suite into a powerful design tool for a general class of polymers. The inclusion of greater numbers of synthetically compatible oligomer scaffolds would allow the design of combinatorial molecules that functionally exceed what is capable by the biopolymers of peptides and nucleic acids. The current implementation as described above is fully compatible with combining various backbones into one molecule such as 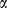-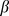-peptide hybrids or peptoid–OOP hybrids. We therefore anticipate the ROSETTA framework to be a useful tool in creating novel polymer designs for a variety of applications.

## Supporting Information

File S1 **OOP Pre ResidueType patch file.** Complete example of OOP patch file.
(TXT)

File S2 **Peptoid NPhe ResidueType parameter file** Complete example of Peptoid parameter file.
(TXT)

Movie S1
**Movie demonstration of OopPuckMover functionality.** Special movers are often necessary to implement which allow proper sampling of noncanonical backbone conformations. The movie shows the steps necessary to change the conformation of an OOP ring from the half-chair to the boat conformation. The OopPuckMover first alters the 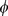 and 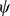 angles and then, second, updates the hydrogens into proper alignment. Gray spheres represent virtual atoms.(MOV)
